# Influence of Co-Catalysts and Polymerization Conditions on Properties of Poly(anhydride-*alt*-epoxide)s from ROCOP Using Salen Complexes with Different Metals

**DOI:** 10.3390/polym11071222

**Published:** 2019-07-22

**Authors:** Matteo Proverbio, Nella Galotto Galotto, Simona Losio, Incoronata Tritto, Laura Boggioni

**Affiliations:** Institute for Macromolecular Studies, National Research Council, Via A. Corti, 12, 20133 Milan, Italy

**Keywords:** polyesters, ROCOP, high glass transition temperature, high molecular weight

## Abstract

Cyclohexene oxide (CHO) and phthalic anhydride (PA) have been reacted in the presence of commercial salen–type complexes with different metals Cr (**1**), Al (**2**), and Mn (**3**) in combination with 4-(dimethylamino) pyridine (DMAP), bis-(triphenylphosphorydine) ammonium chloride (PPNCl) and bis-(triphenylphosphoranylidene)ammonium azide (PPNN_3_) as co-catalysts to obtain alternating poly(PA-*alt*-CHO)s by ring-opening copolymerization (ROCOP). The effect of different reaction conditions (pre-contact between catalyst and co-catalyst, polymerization time) on the productivity, molecular weight and glass transition temperature has been evaluated. By using a 24 h pre-contact, the aliphatic polyesters obtained were characterized by high molecular weight (*M*_n_ > 15 kg/mol) and glass transition temperature (*T*_g_) up to 146 °C; the more sustainable metals Al and Mn in the presence of PPNCl give comparable results to Cr. Moreover, biodegradability data of these polyesters and the study of the microstructure reveal that the biodegradability is influenced more by the type of chain linkages rather than by the molecular weight of the polyesters.

## 1. Introduction

**Scheme 1:**
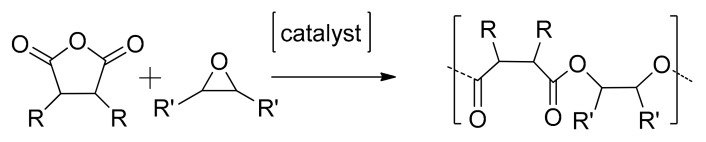
Ring opening copolymerization (ROCOP).

Aliphatic polyesters (PEs) are an appealing class of polymers used in a range of applications such as biomedical devices and bulk packaging owing to their excellent properties and general biocompatibility [1,2]. They are typically synthesized by ring-opening polymerization (ROP) of lactones and lactides [3,4,5], an excellent controlled polymerization route, which gives polymers with relatively low glass transition temperatures. Alternating ring-opening copolymerization (ROCOP) of epoxides and cyclic anhydrides (Scheme 1) is becoming an attractive method for the synthesis of PEs [6,7,8,9,10,11,12,13,14,15]. It represents an alternative chain-growth route to polyesters with respect to the ring-opening polymerization (ROP) of lactones and an opportunity to broaden the range of materials produced and to overcome some of the limitations of ROP. In particular, the properties of the ensuing materials, including the thermal properties (glass transition temperature (*T*_g_) and thermal decomposition temperature), can be tuned by changing the epoxide or cyclic anhydride. For example, low *T*_g_ values can be obtained by monomers with long side chains or using monocyclic epoxides and anhydrides [8,16]. Conversely, high *T*_g_ values are favored by rigid backbones, showing the importance of selecting more rigid monomer combinations such as bi- or tricyclic monomers and monomers bearing an aromatic group [9,10,11,12,13,14,17,18].

However, one of the major challenges faced with ROCOP is that the PEs synthesized via this technique are typically of low number average molecular weight (*M*_n_), that renders poor mechanical and thermal properties and thus limits their applications.

In recent years, numerous organometallic catalysts have been developed for epoxide/anhydride copolymerization, including magnesium [11], aluminum [7,12,13,19,20,21,22], chromium [8,12,15,21,22,23,24,25,26], manganese [12,22,23,27,28], iron [7,29], cobalt [12,15,20,21,22,23], zinc complexes [8,9,11] and heterodinuclear polymerization catalyst [30,31,32,33], many of which showed markedly higher activity with the addition of a nucleophilic co-catalyst [18,29]. Duchateau and coworkers [34] investigated the ROCOP of cyclohexene oxide (CHO) with succinic anhydride (SA), phthalic anhydride (PA), and cyclopropane-1,2-dicarboxylic acid anhydride in bulk and in solution by using different salen or salphen metal complexes such as those of Al, Cr and Co, along with several co-catalysts. The most reactive substrate was PA, which yielded a polyester with the highest *M*_n_ value by using a combination of salphen Cr(III) complex catalyst and bis(triphenylphosphine)iminium chloride (PPNCl) as co-catalyst but no information on thermal properties has been reported. Recently, metal-free initiators for the copolymerization of epoxides with anhydrides are being explored [35,36]. A series of dinuclear complexes, in which two iron(III) amino triphenolate moieties are bridged by a phenylene backbone were synthesized by Jiang [37] for the alternating copolymerization of CHO/PA in the presence of PPNCl with good *M*_n_ value (33 kg/mol). However, research efforts in the synthesis of polyesters by ROCOP with high *T*_g_ and suitable molecular weight by using commercial catalysts are still necessary.

In this study, an investigation on the influence of co-catalysts and polymerization conditions on alternating ROCOP of CHO and PA using commercial salen complexes with different metals is reported. The polyesters were characterized by size exclusion chromatography (SEC), differential scanning calorimetry (DSC) and nuclear magnetic resonance (NMR). This research work offers access to PEs with good thermal and molecular weight properties suitable for practical applications. Moreover, the biodegradability data of selected poly(anhydride-*alt*-epoxide)s are reported.

## 2. Materials and Methods

### 2.1. Materials

Phthalic anhydride (PA), cyclohexene oxide (CHO), 4-(dimethylamino) pyridine (DMAP), bis-(triphenylphosphorydine) ammonium chloride (PPNCl), (R-R)-N,N′-bis (3,5-di-tert-butylsalicylidene)-1,2-cyclohexanediaminochromium(III) chloride, (R-R)-N,N′-bis (3,5-di-tert-butylsalicylidene)-1,2-cyclohexanediaminomanganese(III) chloride were purchased from Sigma-Aldrich, Milan, Italy. (R-R)-N,N′-bis (3,5-di-tert-butylsalicylidene)-1,2-cyclohexanediaminoaluminum chloride was purchased from Strem Chemicals (Newburyport, MA, USA). Cyclohexene oxide and dichloromethane (CH_2_Cl_2_) were dried over CaH_2_, distilled and stored on 4 Å molecular sieves under nitrogen. Phthalic anhydride was recrystallized from dichloromethane prior to use. Bis-(triphenylphosphorydine) ammonium chloride was dissolved in dichloromethane and precipitated in diethyl ether ((C_2_H_5_)_2_O) twice. 4-(dimethylamino) pyridine was double recrystallized from toluene. The co-catalyst bis-(triphenylphosphorydine) ammonium azide has been synthesized according to literature procedures [24]. All manipulations were performed under an inert atmosphere or in a nitrogen-filled MBraun (M. BRAUN INERTGAS-SYSTEME GMBH (Garching, Germany)) glovebox unless stated otherwise.

### 2.2. Synthesis

Bulk Polymerization. 

In a glove box, a 10 mL crimp cap vial equipped with a stirring bar was charged with a mixture of catalyst, co-catalyst, epoxide and anhydride with a ratio of 1:1:250:250. 

Polymerization in Solvent (No Pre-contact Step). 

In a glove box, a 10 mL crimp cap vial equipped with a stirring bar was charged with a mixture of catalyst, co-catalyst, epoxide, anhydride and 1 mL of toluene with a ratio of 1:1:250:250. 

Polymerization in Solvent with Pre-contact step. 

In a glove box, in a 10 mL crimp cap vial equipped with a stirring bar a mixture of catalyst and co-catalyst was charged in the presence of 1 mL of toluene and keep stirring for 1 h or 24 h (pre-contact step). Then, the epoxide and the anhydride were added. The ratio between catalyst: co-catalyst: epoxide: anhydride was 1:1:250:250.

Then, the vial was placed in an aluminum heating block mounted on top of a stirrer/heating plate. At the end of the polymerization the crude product was precipitated twice in methanol and collected after filtration through a 0.45 µm Nylon filter. All the analyses were performed on purified sample. Yield (%) was calculated as $\frac{\mathrm{yield}\;\left(g\right)}{\mathrm{gCHO}+\mathrm{gPA}}*100$.

### 2.3. Methods

The copolymers were weighed in a 5 mm NMR tube and dissolved in CHCl_3_. The spectra were recorded on a Bruker Avance 400 instrument (400 MHz (^1^H); 100.58 MHz (^13^C); pulse angle = 12.50 ms; acquisition time = 0.94 s; delay = 16 s). The probe head was pre-equilibrated at a fixed temperature of 35 °C.

Differential scanning calorimetry (DSC) analysis was performed on a Perkin Elmer DSC 8000 instrument using cyclic heating and cooling rates of 20 °C per minute and heated from 20 to 200 °C. The values of glass transition temperature *T*_g_ were recorded during the second thermal cycle.

Molar mass analysis was performed using about 12 mg of polymer in THF stabilized with 0.025% BHT (butylated hydroxytoluene) at 35 °C by a size exclusion chromatography (SEC) system from Waters W600 (Millford, MA), equipped with a differential refractometer Waters 410. The column set was Agilent 3 PL GEL (Polypore, Oligopore, 50 Å).

Biodegradability have been determined by respirometric biochemical oxygen demand (BOD) Oxitop method based on very accurate automatic pressure measurement in a closed bottle. When organic matter biodegrades, it demands a certain amount of oxygen. When oxygen is consumed, pressure falls and at the same time carbon dioxide is produced. The system consists of an OxiTop-C measuring head, an OxiTop Controller OC 100, capable of handling up to 120 warheads, and an inductive stirring system. Screwing in the OxiTop-C measuring head, like a “cover”, on the special dark glass bottle, it detects the pressure in the head space, using a small transducer connected to a microprocessor. The bottle was placed on a magnetic stirrer suitable for being introduced into an incubator at the set temperature. The sample volume was chosen based on the presumed BOD (biochemical oxygen demand) value, considering that a too large measuring range will lead to inaccurate results. The OxiTop^®^ respirometric system has a special rubber housing inside where NaOH tablets that react with CO_2_ are placed. The removal of CO_2_ from the gas phase led to a decrease in the pressure of the system that was recorded. By means of suitable calculations, the OxiTop-C measuring heads converted the measurement of the pressure variation directly into mg/L of consumed O_2_.

## 3. Results

**Scheme 2:**
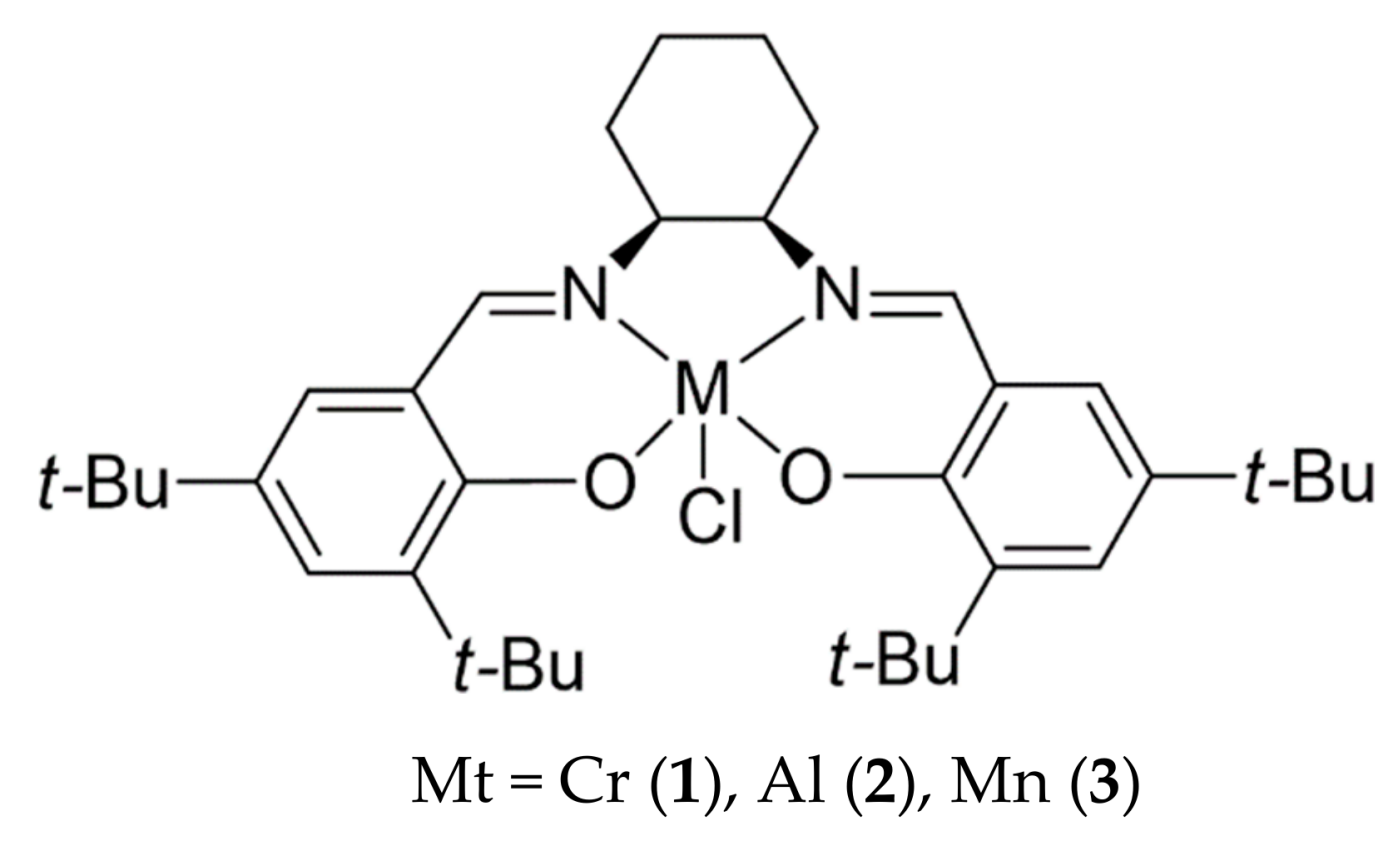
General structure of (salen)MtCl catalysts utilized for the copolymerization reactions.

**Scheme 3:**
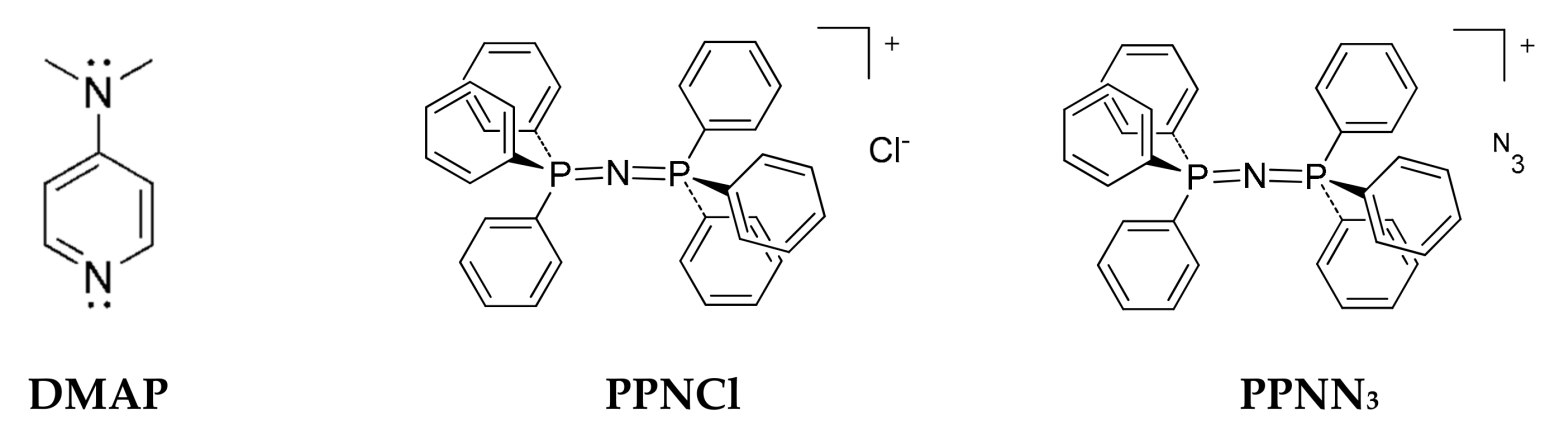
Structures of co-catalysts utilized for the copolymerization reactions.

To understand the effect of the polymerization conditions to obtain industrially processable alternating poly(anhydride-*alt*-epoxide)s with *M*_n_ > 11,000 g/mol and *T*_g_ > 80 °C, a series of copolymerizations of CHO with phthalic anhydride wa performed by the salen-type complexes of three different metals Cr (**1**), Al (**2**), and Mn (**3**) (Scheme 2) in combination with 2-dimethylaminopyridine (DMAP), and two onium salts with a bulky cation and two different anions (PPNCl and PPNN_3_) as co-catalysts (Scheme 3). Commercially available salen complexes were selected, the chromium complex **1** is one of those most studied in ROCOP and in particular was used by Duchateau for the ROCOP of CHO and anhydrides [34]. The aluminum and manganese catalysts **2** and **3**, respectively, were selected as possible more sustainable alternatives. The DMAP and PPNCl co-catalysts investigated are also commercially available. In particular, the neutral Lewis base DMAP is one of the most studied and is taken as reference. PPNX are well-known onium salts: PPNCl is commercially available and in combination with salphen Cr complex resulted in the most effective in CHO and PA copolymerization; PPNN_3_ is reported as one of the most effective co-catalysts in CO_2_ and epoxide copolymerization [38].

### ROCOP of CHO Using Complexes ***1**–**3*** in Presence of Different Co-Catalysts

Initially, the copolymerizations of cyclohexene oxide (CHO) with phthalic anhydride (PA) by (salen)MtCl catalysts **1**, **2** and **3** and DMAP and PPNCl as co-catalysts were performed at 110 °C in bulk without pre-contact step between catalyst and co-catalyst, in polymerization conditions reported in the literature [34]. The results are shown in Table 1.

In our hands, the viscosity of the system increased rapidly especially with catalyst **1** and therefore the reactions were stopped after a short time. Yields were high, catalyst **1** resulted to be the most active one, and in general, PPNCl resulted to be the best co-catalyst. The resulting polyesters were characterized by ^1^H NMR, SEC and DSC. A relatively high amount of ether linkages (see ^1^H NMR results below) was observed under these conditions. The measured numbers average molecular weight *M*_n_ ranged from 1700 to 2500 g/mol. Interestingly, even though molar mases were low probably due to the short polymerization time and high viscosity, the *T*_g_ values were higher than 135 °C, and for copolymers by catalyst **1** *T*_g_ values of 141 °C were recorded.

Successively, with the purpose to increase the molecular weights, a series of copolymerization of CHO and PA were performed in solution by adding 1 mL of toluene in order to reduce the viscosity of the reaction medium. The copolymerizations were carried out in the presence of catalyst **1** and DMAP and as expected, the presence of a small amount of solvent decreases the viscosity of the reaction medium, and the molecular weight of CHO/PA copolymers increases up to 9500 g/mol.

Then, to make homogeneous comparisons in all copolymerizations a pre-contact step between catalysts and co-catalysts was introduced since PPNX salts are insoluble or very sparingly soluble in epoxides at room temperature. Darensbourg [39] reported that for the copolymerization of CHO and CO_2_ a pre-contact step seems to be a key factor in the copolymerization reaction. The catalysts and the different co-catalysts were dissolved in toluene and stirred for 1 h at room temperature (pre-contact step), upon removing the solvent under vacuum, cyclohexene oxide was added with stirring. In Table 2, Table 3 and Table 4 the results of the copolymerizations performed with catalysts **1**, **2** and **3**, respectively, with different co-catalysts at different reaction times, are listed.

The comparison of entries in Table 2 shows in a glance that the pre-contact step was crucial: high values of *M*_n_ for the copolymers prepared in solution and in the presence of the pre-contact step as well as increase in the *T*_g_ values were obtained. In general, good yields were obtained with the three different co-catalysts although the catalytic system **1**/PPNCl gave higher productivity, especially at short polymerization time. A prolonged reaction time (24 h) does not seem to further affect the performance of catalyst **1** with the three co-catalysts.

Regarding the aluminum-based catalyst **2** (Table 3) and the manganese-based catalyst **3** (Table 4), they seemed less promising than the chromium one in terms of productivity at short polymerization time. Conversely, a prolonged polymerization time positively affected the productivity as well the molecular weight and *T*_g_s. Molar masses, yields and *T*_g_ compared well with those obtained with the chromium based complex **1**. PPNCl proved to be the best co-catalyst for the three catalytic precursors.

In addition, we investigated the effect of the pre-contact time on the CHO/PA copolymerizations. A series of additional experiments using a pre-contact time of 24 h and a polymerization time of 1 h was also conducted. The obtained results are compared in Table 5.

For catalyst **1** there was no great performance difference by varying the pre-contact time from 1 to 24 h for the same reaction time (1 h), or using a long pre-contact time (24 h) or a long polymerization time (24 h, see entries 63 vs. 37) for the three different co-catalysts. By using the catalysts **2** and **3**, appreciable differences in the performances have been obtained by varying the pre-contact time from 1 to 24 h for the same reaction time (1 h), while small differences are found by exchanging 1 to 24 h of pre-contact with 24 and 1 h of polymerization time differences. It is worth noting that with 24 h of pre-contact and 1 h of polymerization catalysts **2** and **3**, in terms of activity, molecular weight and thermal properties, compared very well with performances of catalyst **1**. Since this effect was evident also with DMAP as co-catalyst, this indicated that the pre-contact time was important not only because of the solubility of the two phosphonium salts, but also because it facilitated the formation of the active species. This result is important since at higher polymerization time side reactions can occur [19,40,41]. Thus, copolymer microstructure has been investigated by ^1^H NMR.

**Figure 1:**
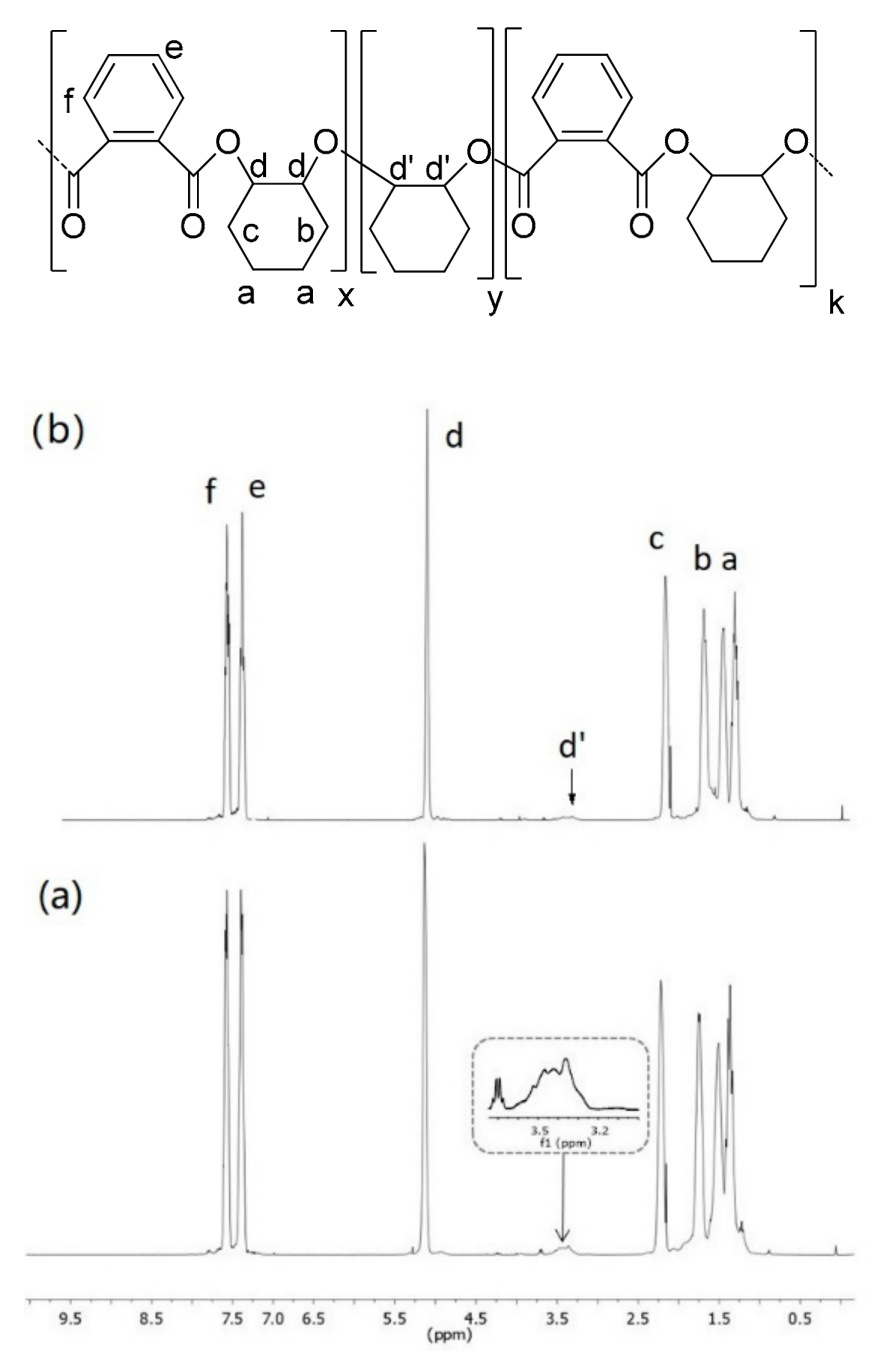
^1^H NMR spectra of poly(CHO-*alt*-PA) obtained with catalyst **1** and PPNCl as co-catalyst with a pre-contact of 1 h and polymerization time of: (**a**) 24 h (entry CHOPA 18) and (**b**) 1 h (entry CHOPA 51).

Indeed, the samples prepared at different pre-contact and polymerization times showed some differences in the microstructure. The ^1^H NMR spectra of the polyesters synthesized with a pre-contact of 1 h and 24 h of polymerization always presented a broad peak between 3.6 and 3.2 ppm corresponding to CHO–CHO ether linkage, as displayed in Figure 1. It seems that the homopolymerization of epoxides could occur as a side reaction when the polymerization is complete [19,40,41].

**Figure 2:**
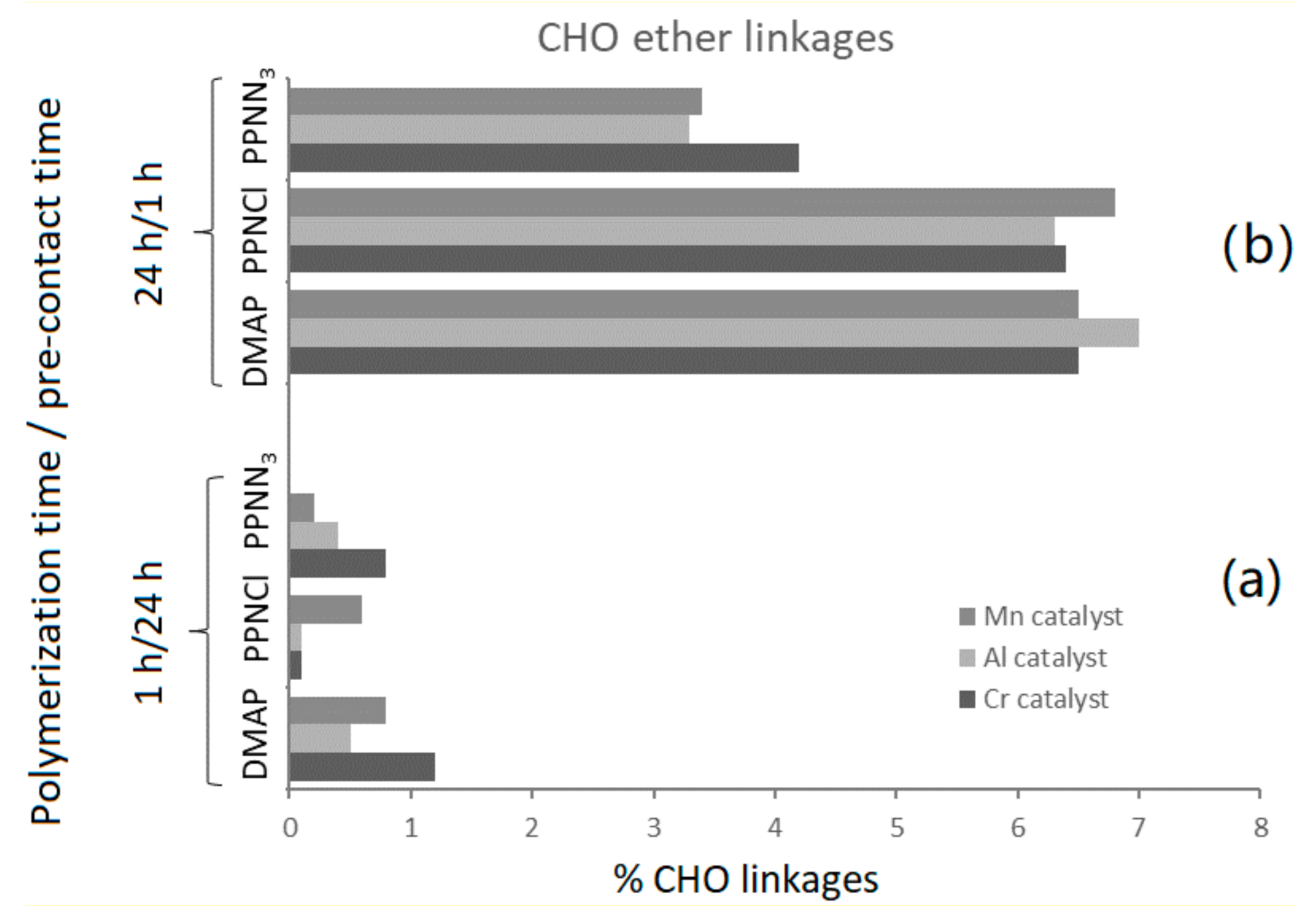
CHO–CHO ether linkage percentage for the different catalytic systems with a polymerization time/pre-contact time of 1 h/24 h (**a**) and of 24 h/1 h (**b**).

This general behavior is well visible in Figure 2, where the CHO ether linkage percentage for the different catalytic systems with a pre-contact of 1 h and different polymerization time is reported. From this point of view, at short polymerization time PPNCl gave the best results with Al and Cr catalysts.

**Figure 3:**
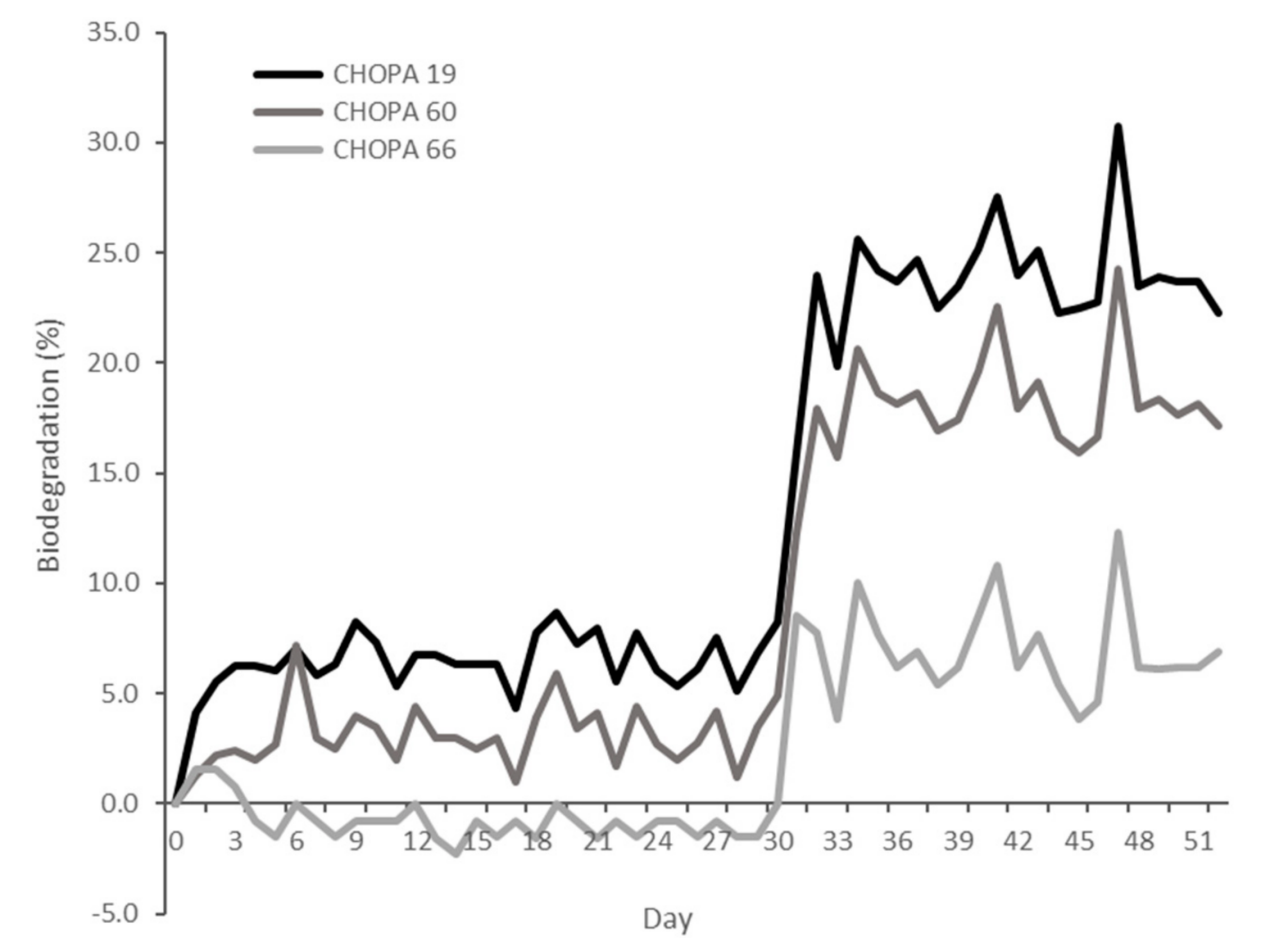
Biodegradation curves for CHO/PA polyesters.

Since biodegradability is one of the interesting properties of these polymers, biodegradability tests were performed according to ISO standards 14,851 (Determination of the ultimate aerobic biodegradability of plastic materials in an aqueous medium) on three polyesters CHOPA 19, CHOPA 60 and CHOPA 66 obtained with catalyst **1** at different polymerization conditions by using the respirometric BOD (biochemical oxygen demand) Oxitop method. CHOPA 19 and CHOPA 60 were obtained in polymerization solution at different pre-contact (1 vs. 24 h) and polymerization times (3 vs. 1 h), showing very similar molecular weight *M*_n_ (17.5 kg/mol for CHOPA 19 and 15.7 kg/mol for CHOPA 60) and ether linkage <7%. CHOPA 66 was obtained in bulk and showed a very low molecular weight (*M*_n_ = 1.6 kg/mol) with an ether linkage >27%. Figure 3 shows the biodegradation curves (average values) as a function of time for the three CHOPA samples and for the microcrystalline cellulose used as reference material. In the curves we can distinguish a lag phase, the biodegradation phase and the plateau. For all three samples the biodegradation phase started after 30 days, that of cellulose after 8 days. The curves of CHOPA 19 and CHOPA 60 were almost overlapping, with a plateau phase close to 32%, well above that of CHOPA 66 which did not reach 12% (Figure 3).

From these data one can deduce that biodegradability was influenced more by the type of chain linkage rather than by the molecular weight of the polyesters. Therefore, the methodology used for polymerization, which influenced polymer microstructure was key in obtaining biodegradable poly(anhydride-*alt*-epoxide)s.

## 4. Conclusions

In conclusion, we examined the influence of different polymerization conditions on the copolymerization of cyclohexene oxide with phthalic anhydride by commercial salen complexes **1**–**3** in combination with DMAP, PPNCl and PPNN_3_. The pre-contact step between catalyst and co-catalyst is a key factor in obtaining polymers with molecular weight > 15 kg/mol and glass transition temperature up to 140 °C in high yields. In general, prolonged polymerization times lead to the formation of large quantities of CHO–CHO ether linkage, which lowers the poly(CHO-*alt*-PA)s biodegradability.

Finally, since with a long pre-contact time, but at short polymerization time, the catalysts **2** and **3** with more sustainable Al and Mn metals in the presence of PPNCl give comparable results to those from the benchmark Cr catalyst, **2** and **3** may be a more sustainable alternative.

## Figures and Tables

**Table 1:** Cyclohexene oxide (CHO)/phthalic anhydride (PA) bulk copolymerization ^a^.

Entry	Catalyst/Co-Catalyst	Time (h)	Yield (%)	Conversion (%) ^b^		Ether Linkages (mol%) ^b^	*M*_n_ (kg/mol)	*Đ*	*T*_g_ (°C)
				CHO	PA				
CHOPA 66	1/DMAP	0.3	74	92	63	27	1.7	3.2	141
CHOPA 69	**1**/PPNCl	0.3	82	87	78	16	1.8	3.0	141
CHOPA 68	**2**/DMAP	1.1	71	78	66	18	2.3	2.5	136
CHOPA 71	**2**/PPNCl	1.1	57	64	53	20	1.9	2.9	137
CHOPA 67	**3**/DMAP	1.0	76	84	71	15	2.4	2.5	139
CHOPA 70	**3**/PPNCl	1.0	81	86	78	16	2.5	2.0	139

^a^ Polymerization conditions: temperature = 110 °C, CHO:PA:cat:cocat = 250:250:1:1, catalyst (20 µmol) and co-catalyst (20 µmol), oxirane (5 mmol) and anhydride (5 mmol). ^b^ Calculated from ^1^H NMR spectra of purified samples.

**Table 2:** CHO/PA copolymerization catalyzed by complex **1** and different co-catalysts ^a^.

Entry	Co-Catalyst	Time (h)	Yield (%)	*M*_n_ (kg/mol)	*Đ*	*T*_g_ (°C)
CHOPA 48	DMAP	1	76	15.5	1.2	147
CHOPA 19	DMAP	3	91	17.5	1.2	146
CHOPA 20	DMAP	24	82	15.4	1.3	146
CHOPA 51	PPNCl	1	90	15.7	1.1	146
CHOPA 17	PPNCl	3	90	16.1	1.2	145
CHOPA 18	PPNCl	24	92	16.8	1.3	145
CHOPA 54	PPNN_3_	1	84	14.8	1.1	146
CHOPA 36	PPNN_3_	3	88	14.2	1.1	147
CHOPA 37	PPNN_3_	24	79	15.2	1.1	146

^a^ Polymerization in solution and with 1 h pre-contact step: solvent = toluene = 1 mL, temperature = 110 °C, CHO:PA:cat:cocat = 250:250:1:1.

**Table 3:** CHO/PA copolymerization catalyzed by complex **2** and different co-catalysts ^a^.

Entry	Co-Catalyst	Time (h)	Yield (%)	*M*_n_ (kg/mol) ^a^	*Đ*	*T*_g_ (°C)
CHOPA 50	DMAP	1	16	4.5	1.36	129
CHOPA 23	DMAP	3	66	10.4	1.47	136
CHOPA 24	DMAP	24	85	14.1	1.24	143
CHOPA 53	PPNCl	1	40	9.4	1.10	133
CHOPA 21	PPNCl	3	84	16.0	1.16	144
CHOPA 22	PPNCl	24	95	15.8	1.25	145
CHOPA 56	PPNN_3_	1	41	8.8	1.10	140
CHOPA 38	PPNN_3_	3	76	12.8	1.17	144
CHOPA 39	PPNN_3_	24	78	9.3	1.24	144

^a^ Polymerization in solution and with 1 h pre-contact step: solvent = toluene = 1 mL, temperature = 110 °C, CHO:PA:cat:cocat = 250:250:1:1.

**Table 4:** CHO/PA copolymerization catalyzed by complex **3** and different co-catalysts ^a^.

Entry	Co-Catalyst	Time (h)	Yield (%)	*M*_n_ (kg/mol)	*Đ*	*T*_g_ (°C)
CHOPA 49	DMAP	1	14	4.5	1.31	144
CHOPA 27	DMAP	3	74	12.0	1.17	/
CHOPA 28	DMAP	24	96	14.0	1.27	/
CHOPA 52	PPNCl	1	35	7.8	1.21	137
CHOPA 25	PPNCl	3	86	14.9	1.15	144
CHOPA 26	PPNCl	24	92	15.1	1.26	144
CHOPA 55	PPNN_3_	1	39	7.5	1.19	136
CHOPA 40	PPNN_3_	3	68	12.6	1.20	138
CHOPA 41	PPNN_3_	24	81	15.7	1.22	144

^a^ Polymerization in solution and with 1 h pre-contact step: solvent = toluene = 1 mL, temperature = 110 °C CHO:PA:cat:cocat = 250:250:1:1.

**Table 5:** CHO/PA copolymerization with different pre-contact times ^a^.

Entry	Catalyst/Co-Catalyst	Polym. Time (h)	Pre-Contact Time (h)	Yield (%)	*M*_n_ (kg/mol)	*Đ*	*T*_g_ (°C)
CHOPA 48	**1**/DMAP	1	1	76	15.5	1.20	147
CHOPA 57	**1**/DMAP	1	24	64	16.3	1.20	147
CHOPA 20	**1**/DMAP	24	1	82	15.4	1.30	146
CHOPA 51	**1**/PPNCl	1	1	90	15.7	1.10	146
CHOPA 60	**1**/PPNCl	1	24	90	15.7	1.13	146
CHOPA 18	**1**/PPNCl	24	1	92	16.8	1.30	145
CHOPA 54	**1**/PPNN_3_	1	1	84	14.8	1.10	146
CHOPA 63	**1**/PPNN_3_	1	24	89	15.5	1.10	143
CHOPA 37	**1**/PPNN_3_	24	1	79	15.2	1.10	146
CHOPA 50	**2**/DMAP	1	1	16	4.5	1.36	129
CHOPA 59	**2**/DMAP	1	24	80	14.2	1.13	145
CHOPA 24	**2**/DMAP	24	1	85	14.1	1.24	143
CHOPA 53	**2**/PPNCl	1	1	40	9.4	1.10	133
CHOPA 62	**2**/PPNCl	1	24	92	16.3	1.11	146
CHOPA 22	**2**/PPNCl	24	1	95	15.8	1.25	145
CHOPA 56	**2**/PPNN_3_	1	1	41	8.8	1.10	140
CHOPA 65	**2**/PPNN_3_	1	24	93	16.2	1.20	148
CHOPA 39	**2**/PPNN_3_	24	1	78	9.3	1.24	144
CHOPA 49	**3**/DMAP	1	1	14	4.5	1.31	144
CHOPA 58	**3**/DMAP	1	24	84	15.4	1.13	147
CHOPA 28	**3**/DMAP	24	1	96	14.0	1.27	144
CHOPA 52	**3**/PPNCl	1	1	35	7.8	1.21	137
CHOPA 61	**3**/PPNCl	1	24	98	14.8	1.09	146
CHOPA 26	**3**/PPNCl	24	1	92	15.1	1.26	144
CHOPA 55	**3**/PPNN_3_	1	1	39	7.5	1.19	136
CHOPA 64	**3**/PPNN_3_	1	24	97	14.7	1.20	145
CHOPA 41	**3**/PPNN_3_	24	1	81	15.7	1.22	144

^a^ Polymerization conditions: solvent = toluene = 1 mL, temperature = 110 °C, CHO:PA:cat:cocat = 250:250:1:1.
